# Duration of Symptoms and Association With Positive Home Rapid Antigen Test Results After Infection With SARS-CoV-2

**DOI:** 10.1001/jamanetworkopen.2022.25331

**Published:** 2022-08-03

**Authors:** Lisa A. Cosimi, Christina Kelly, Samantha Esposito, Scott Seitz, Jacquelyn Turcinovic, John H. Connor, Deborah Hung

**Affiliations:** 1 Division of Infectious Diseases, Brigham and Women’s Hospital, Boston, Massachusetts; 2 National Emerging Infectious Diseases Laboratories, Boston, Massachusetts; 3 Department of Microbiology, Boston University School of Medicine, Boston, Massachusetts; 4 Bioinformatics Program, Boston University, Boston, Massachusetts; 5 The Broad Institute of MIT and Harvard, Cambridge, Massachusetts; 6 Center for Computational and Integrative Biology, Department of Molecular Biology, Massachusetts General Hospital, Boston, Massachusetts

## Abstract

This cohort study assesses the duration of symptoms and association with positive rapid antigen test results after SARS-CoV-2 infection.

## Introduction

Current US Centers for Disease Control and Prevention COVID-19 guidance for nonimmunocompromised individuals allows ending isolation after 5 days if the individual is asymptomatic or afebrile with improving symptoms.^1^ Culturable virus, currently the best proxy for transmissibility, is reported after day 5.^2^ It has been proposed that rapid antigen tests (RATs) might assist in determining isolation periods. However, while RATs correlate with culture positivity during early infection,^3,4^ there are minimal data after day 5, when persistent RAT positivity has been reported.^5,6^ We sought to compare rates of RAT positivity, COVID-19 symptoms, and positive viral culture starting day 6 after a COVID-19 diagnosis.

## Methods

This cohort study was approved by the Mass General Brigham institutional review board. All participants provided online written informed consent. Starting on day 6, individuals newly testing positive tests for SARS-CoV-2 completed an online demographic survey, daily symptom logs, and RAT self-testing. Day 0 was the day of positive SARS-CoV-2 test or symptom onset, whichever came first. On day-6, anterior nasal and separate oral swabs were collected from a convenience sample of 17 individuals (42.5%) for viral culture. Details of the cohort and methods are in the Supplement. The study followed the Strengthening the Reporting of Observational Studies in Epidemiology (STROBE) reporting guideline on cohort studies. A *t* test was used to compare means, using 1-tailed *P* < .05 to signify statistical significance; *R*^2^ was used to explore linear associations between independent variables age, time since last vaccination, and cycle threshold value from initial polymerase chain reaction tests with the dependent variable day of first negative RAT result.

## Results

Between January 5 and February 11, 2022, we enrolled 40 individuals (mean [SD] age, 34 [9.5] years; 23 [57.5%] women and 17 [42.5%] men). Of these, 36 (90.0%) had received a primary vaccine series and first booster dose. Details are shown in the Table. None required hospitalization. In this period, 96% to 99% of sequenced isolates in Boston were Omicron BA.1. Only 10 participants (25.0%) had a negative RAT result on day 6, and all had negative results by day 14 (Figure, A). There were no correlations between day of first negative RAT result and age (*R*^2^ = 0), time since last vaccine (*R*^2^ = 0.05), or cycle threshold value at diagnosis (*R*^2^ = 0.03). The mean (SD) day of first negative RAT result in the 7 never-symptomatic participants vs the 33 ever-symptomatic participants was 8.1 (3.0) vs 9.3 (2.4) (*P* = .14). Positive RAT results were frequent (61 of 90 tests [68%]) on days 6 to 14 among individuals reporting no symptoms that same day (Figure, B).

**Table.** Participant Demographic and Clinical Characteristics

Characteristic	Participants, No. (%) (N = 40)
Sex	
Women	23 (57.5)
Men	17 (42.5)
Age, mean (SD), y	34 (9.6)
Race and ethnicity	
African American/Black non-Hispanic	3 (7.5)
Asian	7 (17.5)
Hispanic	5 (12.5)
White non-Hispanic	21(52.5)
Other/multiracial^a^	4 (10.0)
Known recent COVID-19 contact	19 (47.5)
Had received primary COVID-19 vaccine series^b^	40 (100.0)
Had received COVID-19 vaccine booster^b^	36 (90.0)
Symptomatic at time of first positive test	33 (82.5)
Time since most recent vaccination dose, d	
Mean (SD)	58.9 (38.2)
Median (range)	53.5 (−2 to 184)^c^
Time since most recent prior negative COVID-19 diagnostic test, d	
Mean (SD)	7.7 (9.9)
Median (range)	2.0 (1 to 33)
No. of days of symptoms prior to testing positive	
Mean (SD), d	1.2 (1.8)
Median (range), d	1.0 (−1 to 7)
Ct value of positive COVID-19 PCR test (n = 29)^d^	
Mean (SD)	26.5 (4.8)
Median (range)	28.0 (18.0-33.4)

Abbreviations: Ct, cycle threshold; PCR, polymerase chain reaction.

^a^ Other included 4 participants who did not self-identify with the races or ethnicities listed above and included Ashkenazi Jew, Iranian, Middle Eastern, and Latin American.

^b^ Primary vaccine series indicates 2 doses of an mRNA vaccine or 1 dose of Ad26.COV2.S vaccine. Booster indicates a third dose of mRNA vaccine or 1 dose of mRNA vaccine after a single Ad26.COV2.S vaccine.

^c^ One individual received a booster 2 days after testing positive while awaiting result.

^d^ No. includes the Ct values for the 29 participants whose initial positive diagnostic PCR test was performed at the Broad Institute of MIT and Harvard, Cambridge, Massachusetts.

**Figure.**
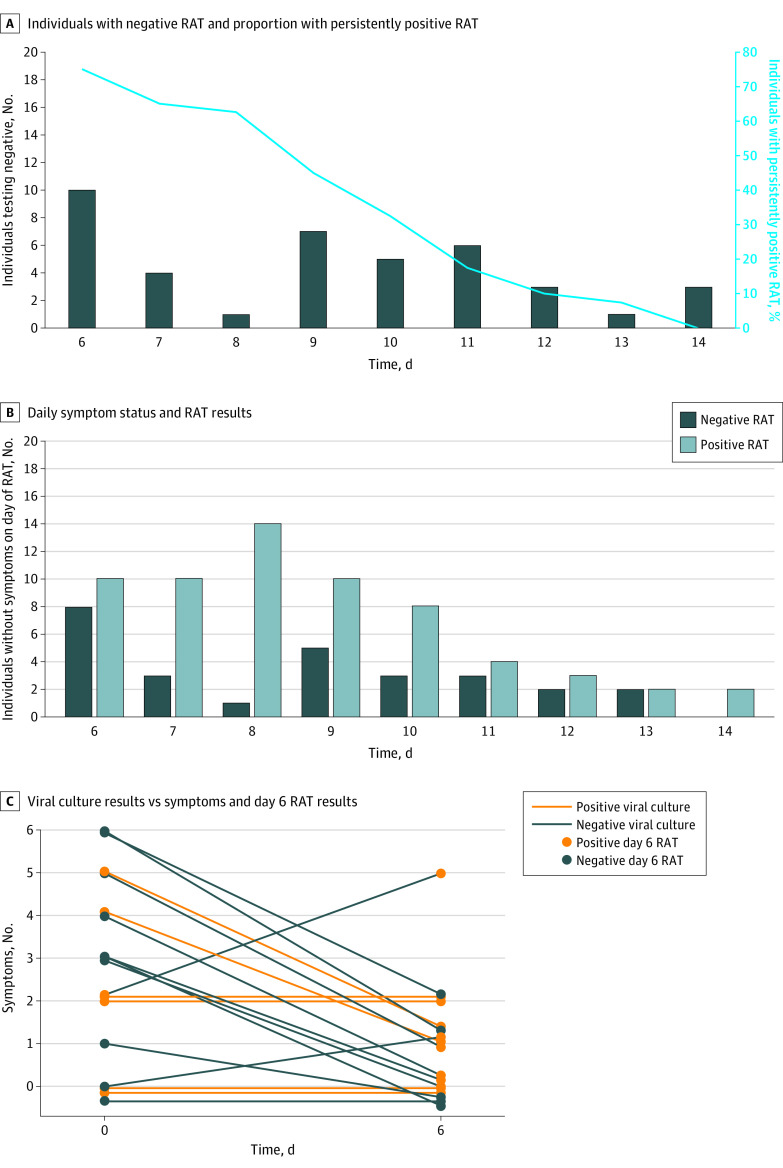
Rapid Antigen Test (RAT) and Viral Culture Results A, Three individuals tested negative after day 10 (days 12, 13, and 14) but had missing testing data on one or more of the previous days. These individuals were censored to negative at earliest possible negative day (day 11 for 2; day 12 for 1). B, A total of 161 RATs were performed in the 40 participants starting on day 6. Among these, 90 tests were performed in individuals who reported no symptoms on the day of the RAT, with 61 having positive and 29 having negative results (negative predictive value, 32%). C, Viral culture results were obtained from 17 individuals. Lines connect numbers of symptoms for each of the 17 on days 0 and 6.

Seventeen individuals were tested for viral culture on day 6, 12 of whom also had a positive RAT result. Of the 12, 6 had positive culture results (5 anterior nasal and 1 oral) (Figure, C). None had positive results from both sites. No individuals with a negative day-6 RAT result had positive cultures. Of the 6 individuals with positive cultures, 2 reported improving symptoms and 2 reported unchanged symptoms, whereas 2 never reported symptoms. Seven of the 9 reporting no symptoms on day 6 (78%) had negative culture results.

## Discussion

In this cohort study of individuals newly diagnosed with COVID-19, 75% continued to have positive RAT results, while 35% had culturable virus on day 6. Everyone with a negative day-6 RAT result had a negative viral culture. However, only 50% of those with a positive RAT result had culturable virus. Acknowledging the caveats of a small cohort of mostly young, vaccinated, nonhospitalized individuals with a presumed Omicron variant and potential variation in self-sampling techniques and lab-based culture methods, these data suggest that a negative RAT result in individuals with residual symptoms could provide reassurance about ending isolation. However, a universal requirement of a negative RAT result may unduly extend isolation for those who are no longer infectious. Meanwhile, a recommendation to end isolation based solely on the presence of improving symptoms risks releasing culture-positive, potentially infectious individuals prematurely, underscoring the importance of proper mask wearing and avoidance of high-risk transmission venues through day 10.

## Notes

Supplement.
eMethods.
