# Error in the Figure

**DOI:** 10.1001/jamanetworkopen.2022.36239

**Published:** 2022-09-23

The Research Letter titled “Duration of Symptoms and Association With Positive Home Rapid Antigen Test Results After Infection With SARS-CoV-2,”^1^ published August 3, 2022, has been corrected to fix an error in the Figure. In Panel B, the bar at day 14 should have been dark blue, indicating negative rapid antigen test results. This article has been corrected.^1^
