# Supplementary material for: Duration of Symptoms and Association With Positive Home Rapid Antigen Test Results After Infection With SARS-CoV-2
Source: JAMA Netw Open. 2022 Aug 3;5(8):e2225331. doi: 10.1001/jamanetworkopen.2022.25331 (PMC9350709; doi:10.1001/jamanetworkopen.2022.25331)
Supplement: Supplement. — eMethods. [file jamanetwopen-e2225331-s001.pdf]

Supplemental Online Content

Cosimi LA, Kelly C, Esposito S, et al. Duration of symptoms and association with positive home rapid antigen test results after infection with SARS-CoV-2. *JAMA Netw Open*. 2022;5(8):e2225331. doi:10.1001/jamanetworkopen.2022.25331

eMethods.

This supplemental material has been provided by the authors to give readers additional information about their work.

© 2022 Cosimi LA et al. *JAMA Network Open.*

eMethods

Description of study cohort: Participants were drawn from a regularly scheduled twice-weekly COVID-19 employee testing program at the Broad Institute of MIT and Harvard. Diagnostic qRT-PCR tests were performed in the Broad's CLIA-certified Clinical Research Processing Platform (CRSP). The FDA EUA approved multi-plex test uses probe sets developed by the CDC targeting two viral gene targets in the Nucleocapsid gene of SARSCoV-2, N1 and N2, and an internal control gene, RNase P.

After release of the U.S. CDC's most recent isolation guidance, the Broad instated a policy whereby all individuals were required to perform COVID testing prior to returning to campus. Individuals who tested positive (either through the employee testing or through an outside test) and wished to return to the Broad's campus prior to 10 days were supplied with at-home RATs from the Broad's environmental health and safety (EHS) group. Per policy, they were allowed to return if asymptomatic and/or afebrile for 24 hours with improving symptoms, and the RAT was negative on or after day 6 with day 0 being the date of their positive diagnostic test or first day of symptoms, whichever came earlier. To be eligible for this study, individuals needed to be an affiliate of the Broad, report a newly positive SARS CoV-2 test to the Broad's EHS group (either through the Broad or through outside testing) and be 18 years or older. All individuals were informed of the study by the Broad's EHS and interested individuals were provided a link to the study site. Individuals followed standard institutional return-to-work protocol regardless of whether they participated. Enrollment, informed consent and all data capture and storage was done online through REDCap (Research Electronic Data Capture), a secure, web-based software platform designed to support data capture for research studies, hosted by MGB. The study was approved by the Mass General Brigham IRB (#2022P000025).

Study Procedures: Beginning on day 6, enrolled individuals performed self-testing with the FDA EUA approved Flowflex™ lateral flow RAT (Acon) - chosen because it is FDA EUA approved for use in asymptomatic individuals[1], uploaded a photo of the results and recorded daily presence or absence of

© 2022 Cosimi LA et al. *JAMA Network Open.*

symptoms (cough, fever, sore throat, difficulty breathing, chest tightness, fatigue, muscle aches, new loss of taste or smell, nausea, vomiting, diarrhea, runny nose, congestion, headache, other) until a negative test resulted. All individuals completed an online survey at entry to record personal demographics including age, sex, race/ethnicity, recent positive COVID-19 contacts, presence and onset of symptoms, COVID-19 vaccination status, and dates of their most recent negative COVID-19 test prior to testing positive. Cycle threshold values were available for the 29 individuals whose initial positive qRTPCR diagnostic test was done at the Broad.

R^2 was used to explore linear associations of the independent variables age, time since last vaccination and SARS-COV-2 PCR cycle threshold value at time of diagnosis to the dependent variable, day of first negative rapid antigen test. A one tailed *t*-test was used to calculate the difference in mean time to first negative RAT between symptomatic and asymptomatic individuals. Statistical significance was determined using $P<.05$.

Viral culture methods: A convenience sampling of observed, self-collection of both anterior nasal (AN) swabs and oral swabs (Sterile Polyester Spun Swab, SteriPack, USA) were collected for viral culture from individuals who were willing to provide samples and where both study staff and participant were available for day 6 testing. Participants were guided through self-collection by trained study team members who travelled to participant homes, with appropriate distancing and PPE. Observed AN swabs were self-collected in the same manner as RATs. Observed oral swabs were collected by asking the participant to cough 3-5 times into their elbow, followed by self-swabbing of each buccal mucosa 10 times and the back of the tongue 10 times until the swab was saturated. Swabs were immediately placed in separate viral transport media tubes (BD™ Universal Transport Media, Becton Dickenson) by the study team member, carried back to the lab and frozen at -20 °C until processing. Viral culture was performed using Caco2 cells engineered to co-express Ace2 and TMPRSS2 which robustly support SARS-CoV-2 replication[2–4]. Caco2 cells were seeded into 24 well dishes (Fisherbrand) in standard culture media (DMEM (Gibco) supplemented with 7% FBS (R and D Systems), 50 ng/mL gentamycin (Gibco)

© 2022 Cosimi LA et al. *JAMA Network Open.*

and 0.25 ug/mL amphotericin B(Gibco) overnight and grown to 60%-90% confluency. The following day 200 uL of viral transfer media from a patient sample was added to the culture media and allowed to incubate at 37 °C. Three days after sample addition, cells were fixed in 10% formalin (Fisherbrand) for 30 minutes at room temperature before removal from the BSL3. Cells were then permeabilized as previously described[5] and analyzed for cytopathic effect (CPE) and evidence of viral replication by indirect immunofluorescence for the presence of SARS-CoV-2. Immunofluorescence was performed using an antibody that recognizes all strains tested of SARS-CoV-2 Nucleoprotein (Cell Signaling Technologies, E8R1L). Positive staining for SARS-CoV-2 growth was marked if cells showed strong immunolabeling in multiple cells and was considered negative if staining was not above mock infected cells. Using this approach, we have regularly cultured from AN and NP swabs with Ct values up to ~32, including Omicron (500 genome equivalents; JHC unpublished results).

<u>eReferences</u>

1. Bekliz M, Adea K, Alvarez C, et al. Analytical sensitivity of seven SARS-CoV-2 antigen-detecting rapid tests for Omicron variant. *medRxiv*. Published online January 1, 2021:2021.12.18.21268018. doi:10.1101/2021.12.18.21268018

2. Chen DY, Khan N, Close BJ, et al. *SARS-CoV-2 Desensitizes Host Cells to Interferon through Inhibition of the JAK-STAT Pathway*. BioRxiv; 2020. doi:10.1101/2020.10.27.358259

3. Wilhelm A, Widera M, Grikscheit K, et al. *Reduced Neutralization of SARS-CoV-2 Omicron Variant by Vaccine Sera and Monoclonal Antibodies*. Infectious Diseases (except HIV/AIDS); 2021. doi:10.1101/2021.12.07.21267432

4. Widera M, Mühlemann B, Corman VM, et al. Surveillance of SARS-CoV-2 in Frankfurt am Main from October to December 2020 Reveals High Viral Diversity Including Spike Mutation N501Y in B.1.1.70 and B.1.1.7. *Microorganisms*. 2021;9(4):748. doi:10.3390/microorganisms9040748

5. Ruedas JB, Arnold CE, Palacios G, Connor JH. Growth-Adaptive Mutations in the Ebola Virus Makona Glycoprotein Alter Different Steps in the Virus Entry Pathway. Dutch RE, ed. *J Virol*. 2018;92(19). doi:10.1128/JVI.00820-18

© 2022 Cosimi LA et al. *JAMA Network Open.*
